# A Novel Human-Infection-Derived Bacterium Provides Insights into the Evolutionary Origins of Mutualistic Insect–Bacterial Symbioses

**DOI:** 10.1371/journal.pgen.1002990

**Published:** 2012-11-15

**Authors:** Adam L. Clayton, Kelly F. Oakeson, Maria Gutin, Arthur Pontes, Diane M. Dunn, Andrew C. von Niederhausern, Robert B. Weiss, Mark Fisher, Colin Dale

**Affiliations:** 1 Department of Biology, University of Utah, Salt Lake City, Utah, United States of America; 2 Department of Human Genetics, University of Utah, Salt Lake City, Utah, United States of America; 3 ARUP Institute for Clinical and Experimental Pathology, University of Utah, Salt Lake City, Utah, United States of America; 4 Department of Pathology, University of Utah, Salt Lake City, Utah, United States of America; University of Toronto, Canada

## Abstract

Despite extensive study, little is known about the origins of the mutualistic bacterial endosymbionts that inhabit approximately 10% of the world's insects. In this study, we characterized a novel opportunistic human pathogen, designated “strain HS,” and found that it is a close relative of the insect endosymbiont *Sodalis glossinidius*. Our results indicate that ancestral relatives of strain HS have served as progenitors for the independent descent of *Sodalis*-allied endosymbionts found in several insect hosts. Comparative analyses indicate that the gene inventories of the insect endosymbionts were independently derived from a common ancestral template through a combination of irreversible degenerative changes. Our results provide compelling support for the notion that mutualists evolve from pathogenic progenitors. They also elucidate the role of degenerative evolutionary processes in shaping the gene inventories of symbiotic bacteria at a very early stage in these mutualistic associations.

## Author Summary

Many insects harbor symbiotic bacteria that perform diverse functions within their hosts. However, the origins of these associations have been difficult to define. In this study we isolate a novel bacterium from a human infection and show that this bacterium is a close relative of the *Sodalis*-allied clade of insect symbionts. Comparative genomic analyses reveal that this organism maintains many genes that have been inactivated and lost independently in derived insect symbionts as a result of rapid genome degeneration. Our work also shows that recently derived *Sodalis*-allied symbionts maintain a significant population of “cryptic” pseudogenes that are assumed to have no beneficial function in the symbiosis but have not yet accumulated mutations that disrupt their translation. Taken together, our results show that genome degeneration proceeds rapidly following the onset of symbiosis. They also highlight the potential for diverse insect taxa to acquire closely related insect symbionts as a consequence of vectoring bacterial pathogens to plants and animals.

## Introduction

Obligate host-associated bacteria often have reduced genome sizes in comparison to related bacteria that are known to engage in free-living or opportunistic lifestyles [1]. This is exemplified by inspection of the genome sequences of mutualistic, maternally transmitted, bacterial endosymbionts of insects, many of which have been maintained in their insect hosts for long periods of evolutionary time [2]. Often these obligate endosymbionts maintain only a small fraction of the gene inventory that is found in related free-living counterparts [3]–[5], indicating that the obligate host-associated lifestyle facilitates genome degeneration and size reduction. At a simple level, the process of genome degeneration in obligate endosymbionts can be viewed as a streamlining of the gene inventory to yield a minimal gene set that is compatible with the symbiotic lifestyle. Genes that have no adaptive benefit are inactivated and deleted as a consequence of mutations that accumulate under relaxed selection at an increased rate in the asexual symbiotic lifestyle as a result of frequent population bottlenecks occurring during symbiont transmission [6].

Although we now have a detailed understanding of the mechanisms and evolutionary trajectory of genome degeneration in ancient obligate insect symbionts, the fundamental question of how these mutualistic associations arise remains to be answered. Studies focusing on insect-bacterial symbioses of recent origin show that closely related bacterial endosymbionts are often found in distantly related insect hosts [7], [8]. This could be explained by the interspecific transmission of symbionts, mediated by parasitic wasps and mites that facilitate the transfer of symbionts between distantly related hosts [9], [10]. Horizontal symbiont transmission could also be mediated by intraspecific mating, as demonstrated in the pea aphid [11]. Another possibility is that symbionts could be acquired *de novo* from an environmental source.

Symbiont acquisition, at least initially, requires the symbiont to overcome or evade the insect immune response. Given that many insects are known to possess a potent immune system that repels invading microorganisms [12], it has been assumed that mutualistic symbionts arise from pathogenic progenitors that have evolved specialized molecular mechanisms to facilitate evasion of the immune response and invasion of insect tissues [2]. In support of this notion, it has been shown that the genomes of recently acquired mutualistic insect endosymbionts maintain genes similar to virulence factors and toxins that are found in related plant and animal pathogens [13]–[18].

In the current study we describe the discovery of a novel human-infective bacterium, designated “strain HS”, isolated from a patient who sustained a hand wound following impalement with a tree branch. Phylogenetic analyses show that strain HS is a member of the *Sodalis*-allied clade of insect endosymbionts. Comparative analyses of the genome sequences of strain HS and related insect symbionts suggest that close relatives of strain HS gave rise to mutualistic associates in a wide range of insect hosts.

## Results

### Isolation and Culture of Strain HS

A 71-year-old male presented to his primary care physician for a routine physical examination three days after sustaining a puncture wound to the right hand. The patient fell and was impaled between the thumb and forefinger by a ∼1 cm diameter branch while removing branches from a dead crab apple tree. Upon presentation the patient denied fever or other constitutional symptoms and had a mild peripheral blood monocytosis (11.8%; reference range = 1.7–9.3%). A palpable cyst was noted in the right hand at the sight of impalement. Warm compresses were applied and cephalexin was prescribed at a dose of 500 mg four times daily for 10 days. The patient was evaluated again three days later due to continuing wound pain. The cyst was drained by aspiration and serosanguineous fluid was submitted for Gram stain and bacterial culture. The Gram stain showed scattered white blood cells, but no bacteria were visualized. A follow-up visit seven days later revealed the presence of an abscess, although the patient was afebrile and without local lymphadenopathy. The abscess was again drained by aspiration and the patient was advised to consult an orthopedic surgeon for evaluation. Subsequent surgery, approximately six weeks later, removed several foreign bodies from the wound and the patient recovered on a second course of cephalexin without incident. Two days after the original cyst aspiration, small numbers of gram negative rods resembling enteric bacteria were isolated on MacConkey agar at 35°C and 5% CO_2_. Colonies were wet, mucoid, variable in size, and slowly fermented lactose. The isolate could not be definitively identified by a manual phenotypic method (RapID ONE, Remel, Lenexa KS) and was misidentified as *Escherichia coli* at 98% confidence by an automated system (Phoenix, BD Diagnostics, Sparks, MD).

### Phylogenetic Analysis of Strain HS

**Figure 1:**
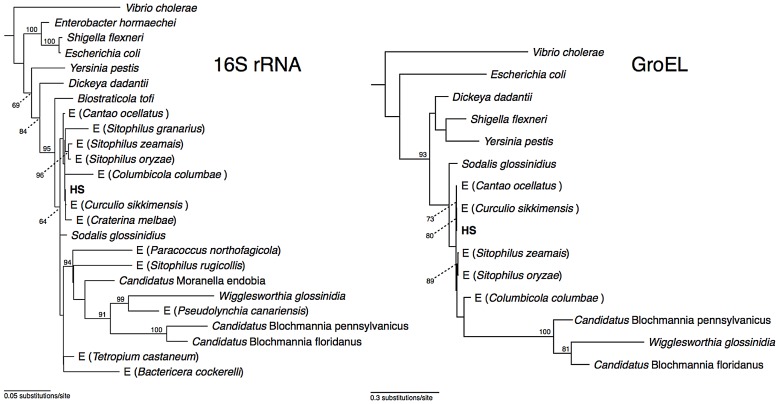
Phylogeny of strain HS and related *Sodalis*-allied endosymbionts and free-living bacteria based on maximum likelihood analyses of a 1.46 kb fragment of 16S rRNA and a 1.68 kb fragment of *groEL*. Insect endosymbionts that do not have proper nomenclature are designed by the prefix “E”, followed by the name of their insect host. The numbers adjacent to nodes indicate maximum likelihood bootstrap values shown for nodes with bootstrap support >70%.

Phylogenetic analysis of 16S rRNA placed strain HS in a well-supported clade comprising *Sodalis*-allied insect endosymbionts sharing >97% sequence identity in their 16S rRNA sequences (Figure 1), which is a commonly used threshold for species-level conservation among bacteria [19]. Aside from strain HS, the closest non-insect associated relative of this clade is *Biostraticola tofi*, which was isolated from a biofilm on a tufa deposit in a hard water rivulet [20]. However, *B. tofi* shares only 96.5% sequence identity in 16S rRNA with its closest insect associated relative (*S. glossinidius*), while strain HS shares >99% sequence identity with the primary endosymbionts of the grain weevils *Sitophilus oryzae* and *S. zeamais* and with recently discovered endosymbionts from the chestnut weevil, *Curculio sikkimensis* and the stinkbug, *Cantao occelatus* [21]–[23]. Analysis of a protein-coding gene, *groEL*, corroborated these findings, confirming that strain HS is a close relative of the grain weevils, chestnut weevil and stinkbug endosymbionts (Figure 1).

### Genome Sequences of Strain HS and Related Insect Symbionts

**Figure 2:**
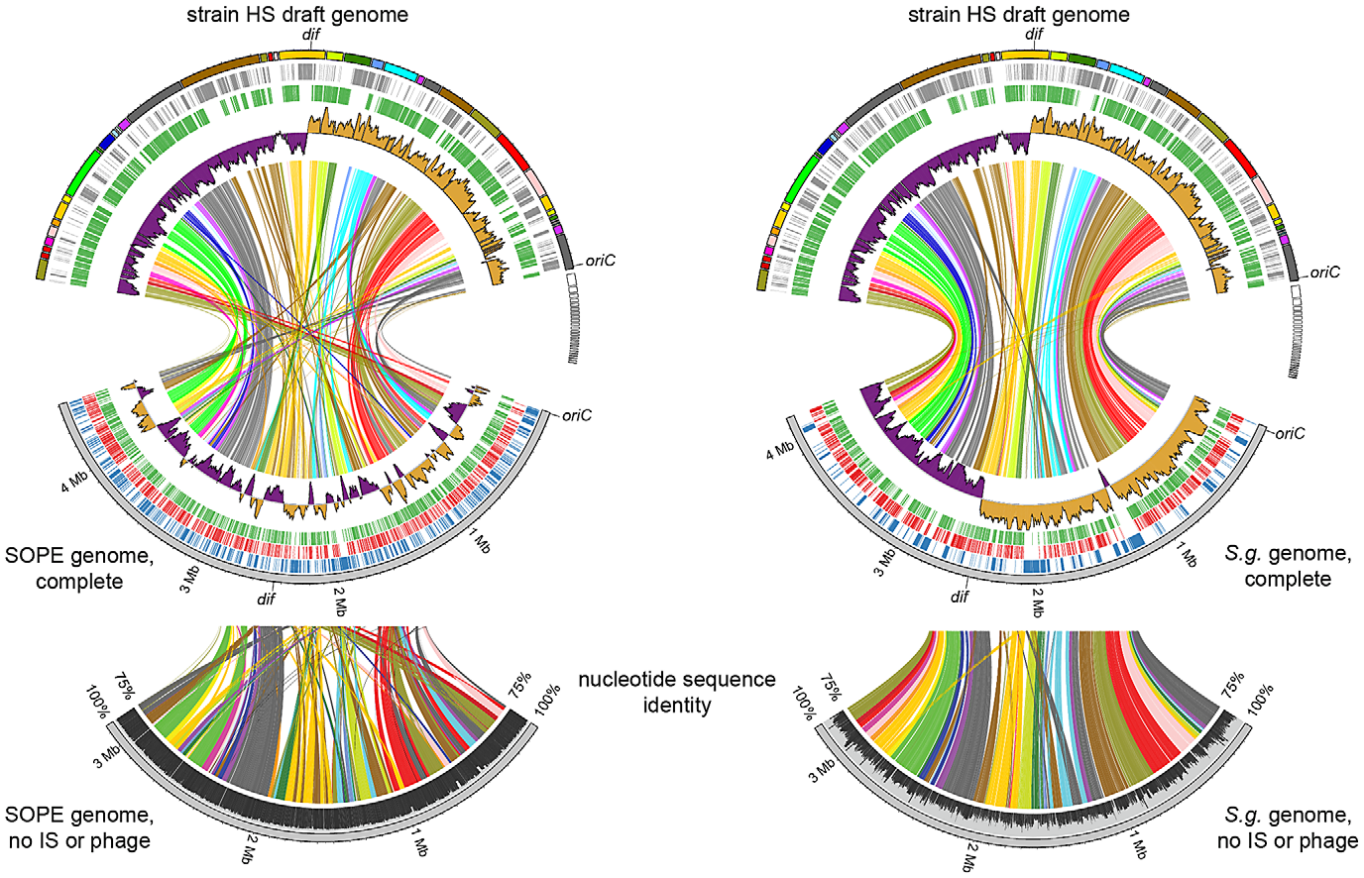
Alignment between strain HS contigs (top) and chromosomes of SOPE (left) and *S. glossinidius* (right). The draft strain HS contigs are depicted in an arbitrary color scheme (outer top ring). Contigs sharing <5 kb synteny with either the SOPE or *S. glossinidius* genome are uncolored. The uppermost plot (colored in purple and orange) depicts G+C skew, based on a 40 kb sliding window. For upper tracks, grey bars depict genes unique to strain HS whereas green bars depict strain HS genes that share orthologs with the aligned symbiont chromosome. For lower tracks, green and red bars represent (respectively) intact and disrupted orthologs of strain HS genes in the insect symbiont genomes, whereas blue bars highlight prophage and IS-element sequences in the insect symbiont chromosomes. Plots of pairwise nucleotide sequence identity are shown in the lower alignment following *in silico* removal of prophage and IS-elements from the SOPE and *S. glossinidius* sequences. Consensus *oriC* and *dif* sequences are labeled to indicate putative origins and termini of chromosome replication.

To compare the genome sequences of strain HS and related *Sodalis*-allied endosymbionts, we aligned a draft sequence assembly of strain HS, comprising a total of 5.15 Mb of DNA in 271 contigs, with the complete genome sequences of the tsetse fly secondary endosymbiont, *S. glossinidius* (4.3 Mb) [24], [25], and the recently completed sequence of *Sitophilus oryzae* primary endosymbiont (SOPE; 4.5 Mb). The resulting alignments (Figure 2) reveal a remarkable level of conservation in gene content and organization between strain HS, *S. glossinidius* and SOPE. To determine if this high level of conservation is simply a consequence of the close evolutionary relationship between these bacteria, we also constructed a whole genome sequence alignment between strain HS and *Dickeya dadantii*, which represents the next most closely related free-living bacterium whose whole genome sequence is available (Figure S1). This alignment shows that strain HS and *D. dadantii* are substantially more divergent in terms of their gene inventories, consistent with the notion that they occupy distinct ecological niches. Considering the alignments between strain HS, *S. glossinidius* and SOPE, it is notable that while the genome sequences of strain HS and *S. glossinidius* display an increased level of co-linearity, the relationship between strain HS and SOPE is predicted to be closer based on the fact that they share a higher level of genome-wide sequence identity (Figure 2). The genome sequences of strain HS and *S. glossinidius* demonstrate a typical pattern of polarized nucleotide composition in each replichore (G+C skew, Figure 2), whereas the SOPE genome has numerous perturbations in G+C skew that must result from recent chromosome rearrangements. These rearrangements likely arose as a consequence of intragenomic recombination events between repetitive insertion sequence (IS)-elements, which are highly abundant in the SOPE genome (Figure S2), and have been documented as a causative agent of deletogenic rearrangements in other bacteria [26]–[28].

**Figure 3:**
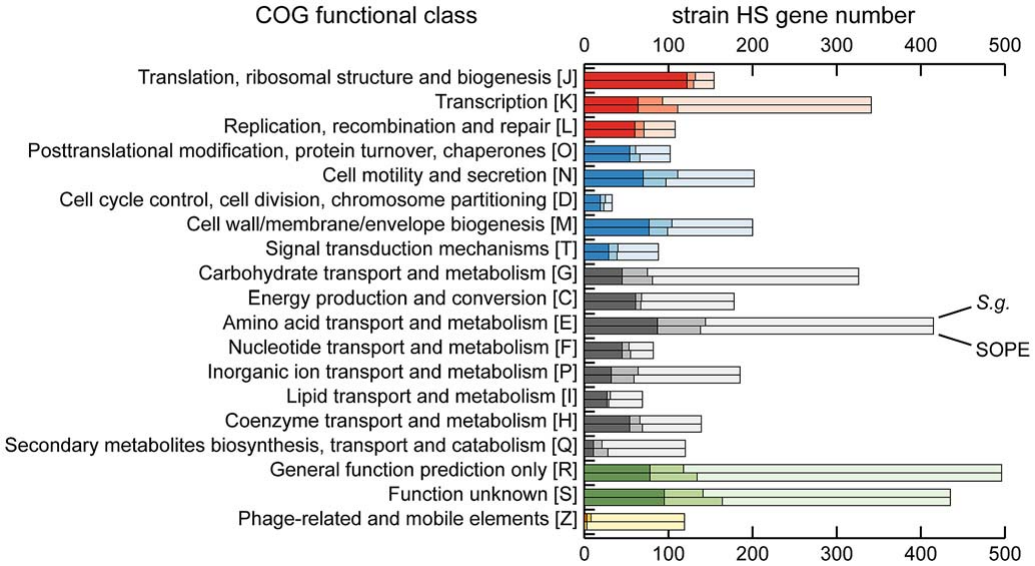
Retention of strain HS orthologs in *S. glossinidius* and SOPE according to COG functional category. The dark shaded component of each bar refers to intact genes retained in both *S. glossinidius* and SOPE. The intermediate shaded component refers to intact genes retained in only *S. glossinidius* (upper bar) or SOPE (lower bar) and the lighter shaded component refers to genes that are either absent or disrupted in both *S. glossinidius* and SOPE. The COG categories are organized in five larger groups with red representing genes involved in information storage and processing, blue representing genes involved in cellular processes and signaling, black representing genes involved in metabolism, green representing genes with poorly characterized functions, and yellow representing components of phages and IS-elements.

Although the gene inventories of strain HS, *S. glossinidius* and SOPE share many genes in common, as expected given their close evolutionary relationship, each bacterium also maintains a fraction of unique genes. In strain HS we identified a total of 1.9 Mb of DNA encoding genes not found in either *S. glossinidius* or SOPE that are classified in a wide range of functional categories (Figure 3). This indicates that strain HS has many unique genetic and biochemical properties, and is consistent with the observation that strain HS, unlike the fastidious and microaerophilic *S. glossinidius* [14], grows under atmospheric conditions on minimal media. In addition, strain HS maintains a number of unique genes sharing high levels of sequence identity with virulence factors found in both animal and plant pathogens, including an Hrp-type effector protein that is characteristically utilized by plant pathogenic bacteria [29] (Table S1). This may be indicative of the ability of strain HS to sustain infection in plant tissues. In comparison with strain HS, the unique fractions of the *S. glossinidius* and SOPE chromosomes are composed almost exclusively of components of mobile genetic elements, including integrated prophage islands and IS-elements. Following excision of these mobile genetic elements *in silico* prior to alignment, the resulting genome sequences of *S. glossinidius* (3.21 Mb) and SOPE (3.15 Mb) represent near-perfect subsets of the strain HS genome (Figure 2), indicating that *S. glossinidius* and SOPE are abridged derivatives of a strain HS-like ancestor.

### Independent Gene Inactivation and Deletion in *S. glossinidius* and SOPE

**Figure 4:**
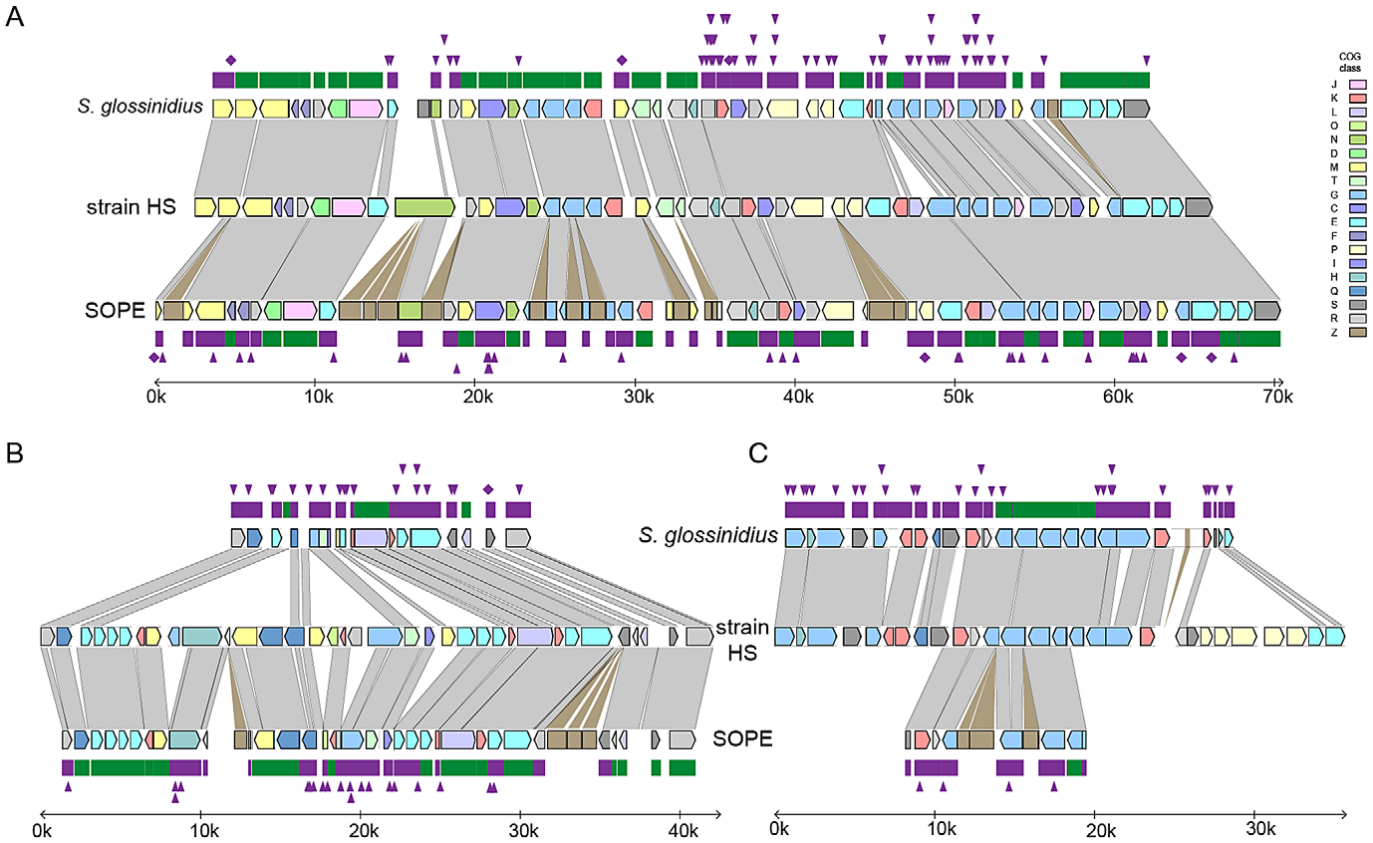
Alignments of three regions of the *S. glossinidius*, strain HS, and SOPE chromosomes. Alignments of three regions of the *S. glossinidius*, strain HS, and SOPE chromosomes, corresponding to SG0948–SG0977 (A), ps_SGL0466–SG0918 (B) and ps_SGL0318–ps_SGL0330 (C) in the most recent *S. glossinidius* annotation [25]. Putative ORFs and intergenic regions are drawn according to scale, oriented according to their inferred direction of transcription and color-coded according to COG functional categories. While all of the depicted strain HS genes have intact reading frames, the status of their orthologs in *S. glossinidius* and SOPE are shown in the outer bars (green = intact, purple = inactivated). Nonsense mutations (premature stop codons) are depicted by purple diamonds, and frameshifting indels are depicted by purple triangles. Light grey connecting bars are syntenic nucleotide alignments, while brown bars illustrate IS-element acquisitions that occur more frequently in SOPE.

To further understand genetic differences between strain HS, *S. glossinidius* and SOPE, we analyzed three genomic regions containing relatively high densities of pseudogenes in both *S. glossinidius* and SOPE (Figure 4). The most notable finding to arise from this comparison is the absence of pseudogenes in the three genomic regions of strain HS. Furthermore, our comparative analysis shows that *S. glossinidius* and SOPE each have a unique complement of pseudogenes. Indeed, even for orthologous genes that have been inactivated in both *S. glossinidius* and SOPE, mutations leading to gene inactivation in each insect symbiont genome are distinct, indicating that gene inactivation and loss took place independently in *S. glossinidius* and SOPE, mostly as a consequence of small frameshifting indels. However, it should also be noted that the reductions observed in the gene inventories of *S. glossinidius* and SOPE are very similar at the level of functional categories, indicating that the insect-associated lifestyle imposes similar constraints on the retention of genes encoding core functions such as replication, transcription, translation and energy generation (Figure 3). In order to determine the number of pseudogenes throughout the genome of strain HS, we performed a manual annotation and careful inspection of the complete draft strain HS sequence assembly. Out of a total of 4,002 intact candidate ORFs identified in the draft annotation (Table S1), only 48 (including phage and IS elements) were found to be translationally frameshifted or truncated by more than 10% of the size of their most closely related orthologs in the GenBank database (Table 1). This finding stands in stark contrast to the gene inventories of both *S. glossinidius* and SOPE, in which pseudogenes represent a substantial fraction of their total genomic coding capacity (Figure 2) [24], [25]. Thus, for both *S. glossinidius* and SOPE, the predominant evolutionary trajectory following obligate insect association involved the inactivation and/or loss of a substantial component of the ancestral (strain HS-like) gene inventory.

**Table 1:** General features of the strain HS, SOPE, and *S. glossinidius* genome sequences.

	Chromosome size	Number of intact genes	Number of pseudogenes	Mobile DNA	G+C content
Strain HS	5.16 Mb^a^	4002 (4364)^b^	48	0.14 Mb	56.73%
SOPE	4.51 Mb	1414	1194	1.36 Mb	56.06%
*S. glossinidius*	4.17 Mb	1355	1376	0.96 Mb	54.69%

The chromosome size of strain HS is estimated based on the combined size of non-redundant contigs in the draft sequence assembly. The number in parentheses indicates the total number of candidate genes identified in strain HS, including representatives that are fragmented in the current assembly.

a Estimated based on current draft assembly.

b Total number of genes identified in strain HS draft genome. Genes containing gaps in the draft assembly were excluded from all comparative analyses.

### Evolution of Pseudogenes in *S. glossinidius* and SOPE

**Figure 5:**
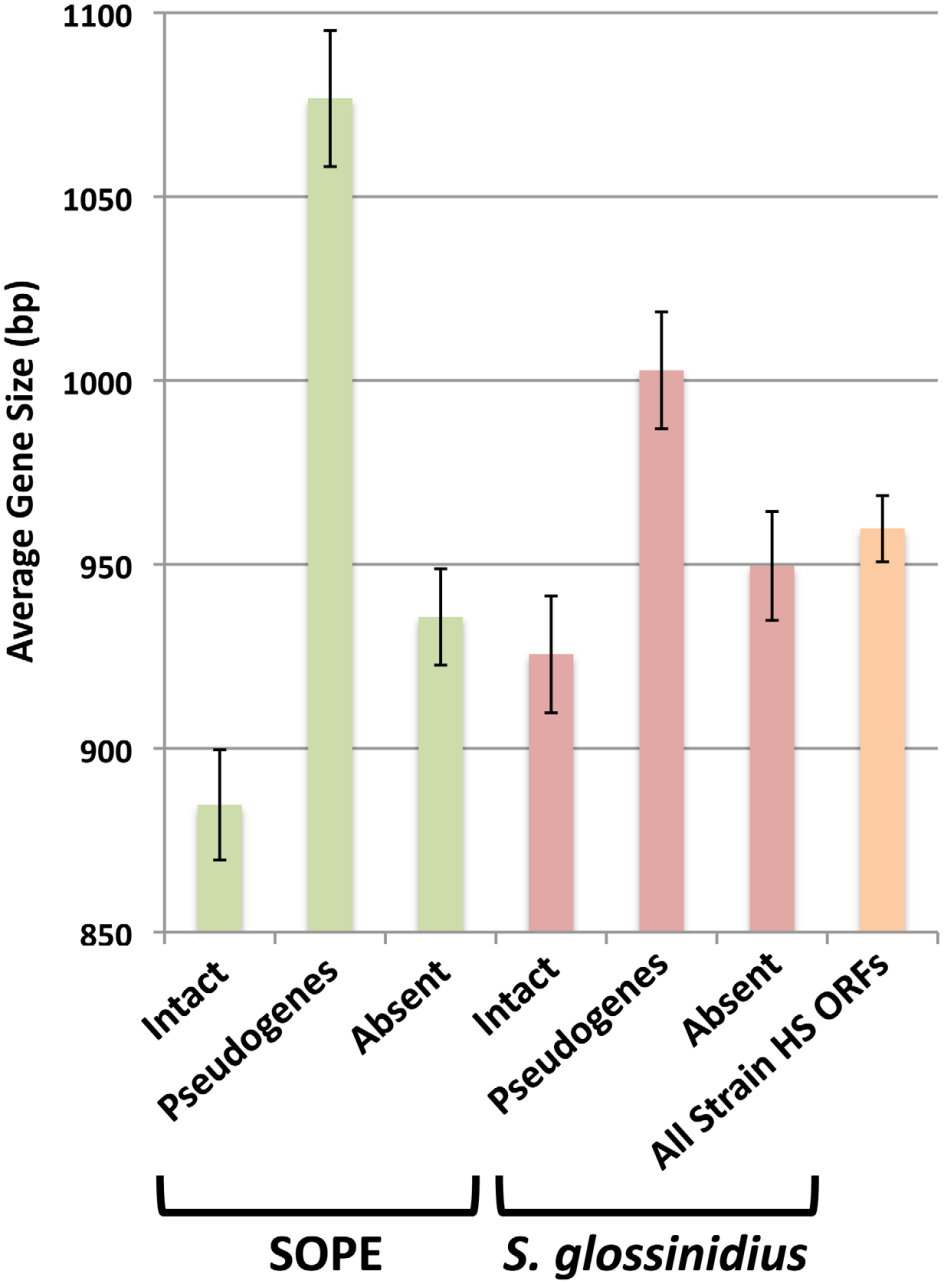
Average size of strain HS orthologs classified as intact, pseudogenized, and absent in SOPE (green) and *S. glossinidius* (red). The average size of all strain HS ORFs is also shown in orange. Error bars depict the standard errors of the mean.

The close evolutionary relationships between strain HS, *S. glossinidius* and SOPE indicate that the respective insect symbioses are recent in origin. This raises the possibility that a subset of selectively neutral genes in the *S. glossinidius* and SOPE genomes have not yet accumulated mutations that lead to disruption of their open reading frames. Such “cryptic” pseudogenes are assumed to have no adaptive benefit in the symbiosis and are expected to accumulate nonsense and/or frameshifting mutations in the future [30]. To determine if the genomes of *S. glossinidius* and SOPE maintain cryptic pseudogenes, we compared the average size of all strain HS genes with the average sizes of strain HS orthologs that are classified either as intact, absent (lost via large deletion) or pseudogenes (visibly disrupted) in the *S. glossinidius* and SOPE genomes (Figure 5). First, it is important to note that the average size of the absent strain HS orthologs in *S. glossinidius* and SOPE is not significantly different from the average size of all strain HS ORFs, indicating that large deletion events are not significantly biased with respect to size. However, in both *S. glossinidius* and SOPE, genes in the pseudogene class were found to have a larger average size in comparison to all strain HS orthologs. Similarly, genes in the intact class were found to have a smaller size in comparison to all strain HS orthologs. This can be explained by the fact that larger genes have an increased likelihood of accumulating at least one disrupting mutation in a given time frame. Based on the same logic, we can infer that the intact gene class contains a subset of smaller, cryptic pseudogenes that have not yet had sufficient time to accumulate any nonsense or frameshifting mutations. Furthermore, since the difference between the average size of intact and disrupted genes is significantly larger in SOPE (192 bases) in comparison to *S. glossinidius* (77 bases), it follows that SOPE likely maintain a larger number of cryptic pseudogenes than *S. glossinidius*.

### Estimating Numbers of Cryptic Pseudogenes in *S. glossinidius* and SOPE

**Figure 6:**
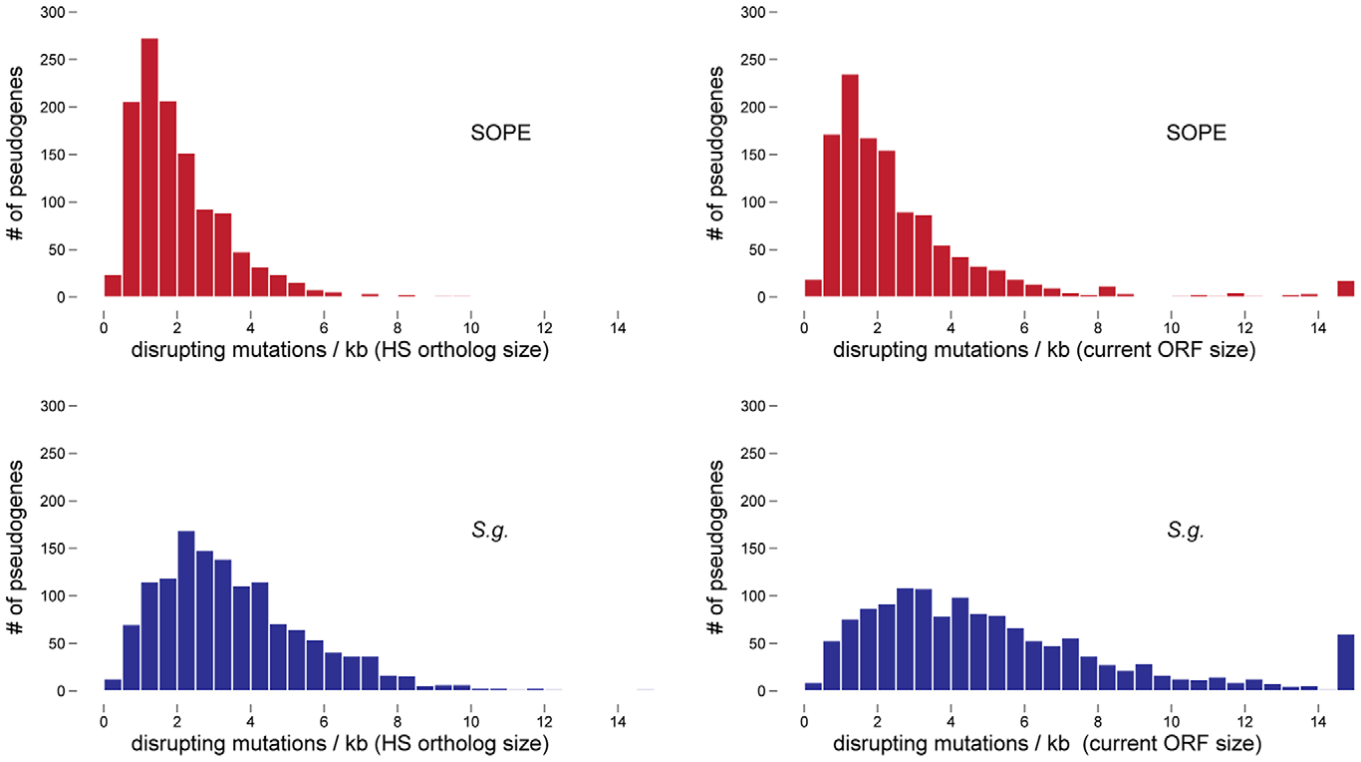
Densities of disrupting mutations in SOPE and *S. glossinidius* pseudogenes. The numbers of frameshifting and truncating indels and nonsense mutations were computed from alignments of strain HS, SOPE and *S. glossinidius* orthologs. Mutation densities were computed according to the original strain HS ORF sizes (left) or the current SOPE or *S. glossinidius* pseudogene sizes (right).

**Figure 7:**
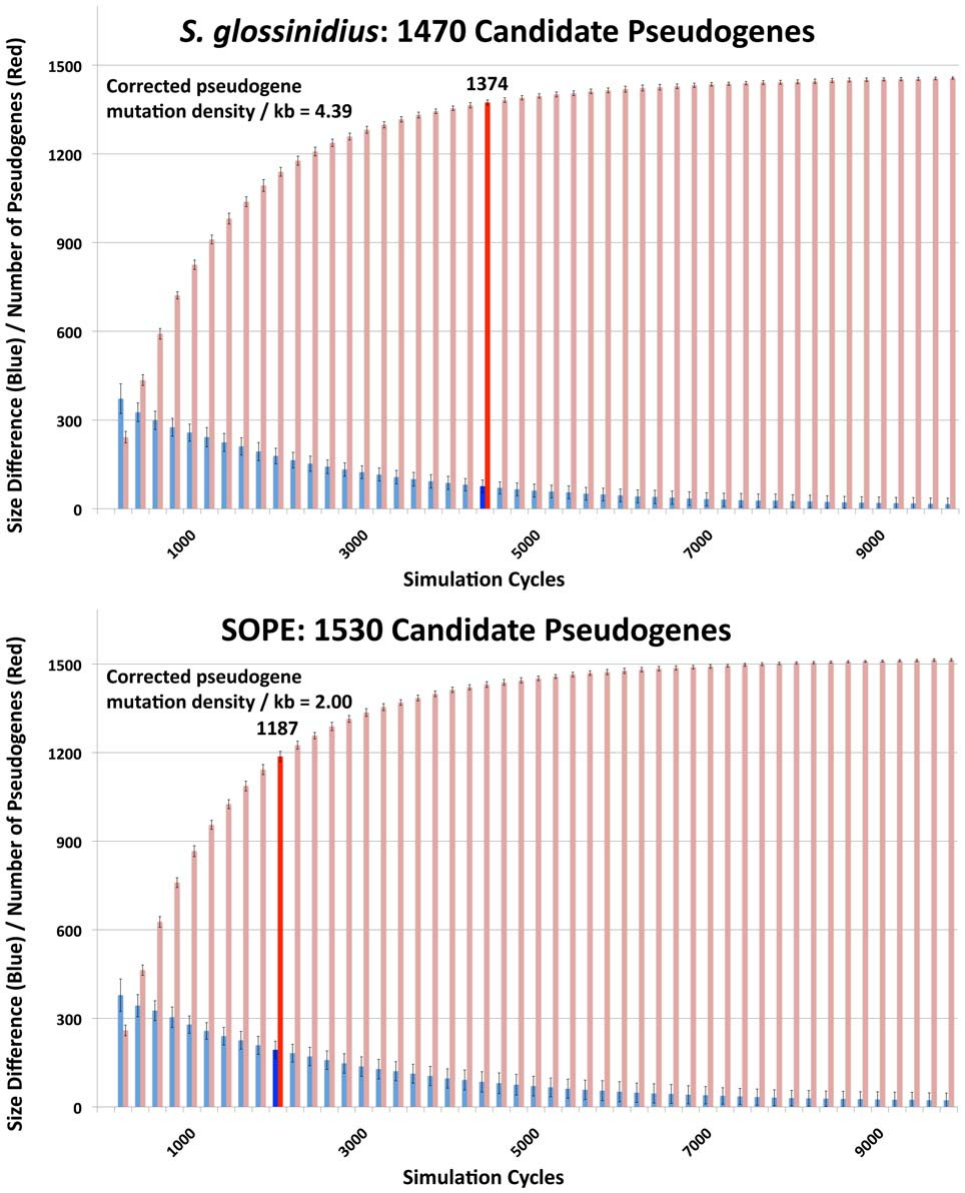
Numbers of cryptic pseudogenes in *S. glossinidius* and SOPE estimated using a Monte Carlo simulation. The simulation was repeated with an increasing number of candidate pseudogenes until estimates of pseudogene number (red) and the size difference between pseudogenes and intact genes (blue) matched empirical values shown in Figure 5 and Table 1, as highlighted by bold bars. The densities of disrupting mutations in *S. glossinidius* and SOPE pseudogenes (which include cryptic pseudogenes) are shown in the upper left inset, corresponding to the data points highlighted in bold.

In a previous study, the numbers of cryptic pseudogenes in the recently derived aphid symbiont, *Serratia symbiotica*, were estimated by extrapolation from a Poisson distribution of disrupting mutations found in existing pseudogenes [30]. The expectation of a Poisson distribution is based on the assumption that the switch to an insect-associated lifestyle leads to the synchronous relaxation of selection on genes no longer required for persistence in an insect host [30]. In the case of both SOPE and *S. glossinidius,* plots of the densities of disrupting mutations in pseudogenes indicate that the data is overdispersed relative to a Poisson distribution (Figure 6). This effect is exacerbated when current ORF sizes are used for the calculation of mutation densities. This results from the fact that large deletions erase any evidence of previous disrupting mutations. In order to estimate the numbers of cryptic pseudogenes in SOPE and *S. glossinidius,* we used a Monte Carlo simulation in which a randomly selected class of candidate pseudogenes, selected from all strain HS genes, was permitted to accumulate random disrupting mutations over time, in accordance with ORF size. In this simulation, both pseudogene counts and size differences between the strain HS orthologs of intact and disrupted *S. glossinidius* and SOPE genes were recorded at regular intervals. The simulation was repeated with an increasing number of neutral genes until the size difference and pseudogene count matched the empirically determined values shown in Figure 5 and Table 1. For *S. glossinidius* and SOPE, matches were obtained when the predicted numbers of genes evolving under relaxed selection reached 1,470 and 1,530, respectively (Figure 7). Thus, although *S. glossinidius* and SOPE are predicted to have almost the same numbers of genes evolving under relaxed selection, the degeneration of pseudogenes is at a more advanced stage in *S. glossinidius*, and SOPE has a larger proportion of neutral genes that have not yet acquired any obvious disrupting changes. Assuming that the relaxation of selection was imposed synchronously at the onset of obligate insect-association, these results suggest that the SOPE-weevil symbiosis originated more recently than the *S. glossinidius*-tsetse fly symbiosis. This is further supported by a comparison of the estimates of corrected mutation density derived from the simulation (Figure 7). While SOPE is estimated to maintain only 2 disrupting mutations/kb of pseudogenes, *S. glossinidius* is estimated to maintain more than twice that density of disrupting substitutions (4.39 disrupting mutations/kb). On a related note, we were unable to utilize *d*N/*d*S ratios to identify cryptic pseudogenes in SOPE or *S. glossinidius*. This is likely due to the fact that stochastic variation resulting from differences in expression level, codon bias and other factors greatly exceeds any signal resulting from a recent relaxation of selection.

### Accelerated Sequence Evolution and Base Composition Bias in SOPE and *S. glossinidius*

**Figure 8:**
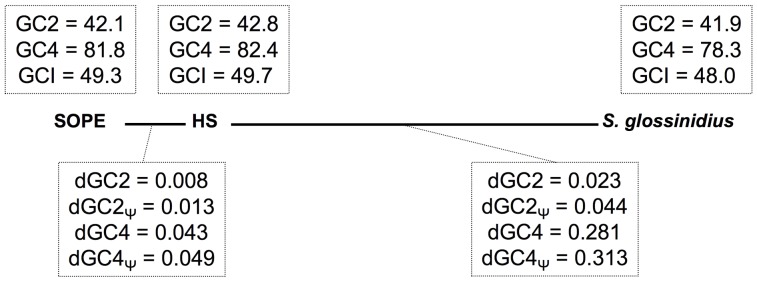
Base composition bias and mutation rates observed in pairwise comparisons between strain HS, *S. glossinidius* and SOPE. The evolutionary relationships between SOPE, strain HS and *S. glossinidius* are depicted by bold lines drawn to scale in accordance with levels of genome-wide divergence at 4-fold degenerate (GC4) sites. Upper boxes show genome-wide GC-percentages at 2^nd^ codon position (GC2), GC4 and intergenic (GCI) sites. Lower boxes depict the number of substitutions per site for intact genes (dGC2 and dGC4) and pseudogenes (dGC2_Ψ_ and dGC4_Ψ_). The data were obtained from pairwise analysis of point mutations in 1,355 intact genes and 1,376 pseudogenes shared between strain HS and *S. glossinidius*, and 1,414 intact genes and 1,194 pseudogenes shared between strain HS and SOPE.

The transition to obligate insect-association is also known to catalyze base composition bias and accelerated polypeptide sequence evolution on the part of the symbiont [31]. The results outlined in Table 1 show that the genomic GC-contents of *S. glossinidius* and SOPE are lower than that of strain HS. However, to avoid any bias arising from the differential gene content of these organisms, we also performed comparative analyses focusing solely on orthologous sequences. This facilitated the comparison of 1,355 intact genes and 1,376 pseudogenes shared between strain HS and *S. glossinidius*, and 1,414 intact genes and 1,194 pseudogenes shared between strain HS and SOPE. Although the symbioses in the current study are anticipated to be relatively recent in origin, comparisons focusing on these shared sequences also show that both *S. glossinidius* and SOPE have reduced GC-contents relative to strain HS (Figure 8). This effect is most notable at 4-fold degenerate (GC4) sites in *S. glossinidius*, which demonstrate the highest levels of sequence divergence and AT-bias in comparison to orthologs from strain HS. Assuming that the onset of AT-bias is coincident with the origin of symbiosis, this further supports the notion that the symbiosis involving *S. glossinidius* is more ancient in origin. It is also notable that the number of substitutions at the 2^nd^ codon position sites of pseudogenes (dGC2, Figure 8) is elevated by approximately the same extent (relative to intact genes) in *S. glossinidius* and SOPE. This implies that pseudogenes have been evolving under relaxed selection for approximately the same proportion of time since each symbiont diverged from strain HS. However, given that sequence divergence at silent sites (GC4) is greater between strain HS and *S. glossinidius*, this again invokes the interpretation that pseudogenes arose earlier in the *S. glossinidius* line of descent. It is also interesting to note that the level of divergence at GC2 sites (dGC2, Figure 8) relative to GC4 sites (dGC4, Figure 8) is greater in SOPE than in *S. glossinidius*. This can be explained by the fact that the pairwise comparison between strain HS and SOPE is expected to capture an increased proportion of mutations that are fixed in the insect-associated phase of life in which selection on polypeptide evolution is anticipated to be more relaxed.

### Mutational Dynamics of Gene Inactivation in SOPE and *S. glossinidius*

Considering only those mutations that have led to gene inactivation, we found that the relative ratios of truncating (large) indels, frameshifting (small) indels and nonsense mutations are similar in SOPE and *S. glossinidius* (Table 2). Inspection of the data reveals that small frameshifting deletions constitute the most abundant class of mutations leading to gene inactivation. However, it should be noted that the effects of large deletions are, for obvious reasons, not captured in our analyses. Another important point is that IS-element insertions appear to have contributed relatively little to the overall spectrum of mutations leading to gene inactivation in SOPE, representing only 10% of the total count. Indeed, the majority of IS-elements in SOPE are located either in intergenic regions or, more commonly, clustered inside other IS-elements. One potential explanation is that IS-element insertions in genic sequences might be more deleterious towards processes of transcription and/or translation in the cell, such that pseudogenes with IS-element insertions are preferentially deleted relative to pseudogenes with nonsense point mutations or small indels. However, it is conspicuous that clustering of IS-elements has also been reported for mobile DNA elements found in eukaryotes, including the MITE elements found in plants [32] and mosquitoes [33], and the Alu and L1 elements found in the human genome [34]. The relative paucity of IS-elements in genic DNA is surprising given the fact that the SOPE genome has such a large number of pseudogenes that provide neutral space for IS-element colonization. However, the inability of IS-elements to occupy this territory can be rationalized as a consequence of an inherited adaptive bias that facilitates the avoidance of genic insertion. This makes sense when considering the perspective of an IS-element residing in a free-living bacterium that has relatively few dispensable genes. It also explains the propensity for IS-elements to insert themselves into the sequences of other IS-elements, because the safety of this approach has already been validated by natural selection. Clearly, in the case of SOPE, when the opportunity arose for expansion into novel territory (i.e. neutralized genic sequences), IS-elements were largely unable to overcome these basic evolutionary directives.

**Table 2:** Allelic spectrum of pseudogene mutations in strain HS orthologs found in SOPE and *S. glossinidius*.

Pseudogenes	Disrupting mutations	Internal insertions	Internal deletions	5′ deletions	3′ deletions	Nonsense mutations^a^	IS elements
SOPE (1194)	2249	19%	34%	7%	11%	19%	10%
*S. glossinidius* (1376)	4316	13%	40%	12%	19%	16%	-

Numbers in parentheses indicate the number of pseudogenes of strain HS orthologs found in the SOPE or *S. glossinidius* genome sequences. Nonsense mutations are classified as point mutations that catalyze the incorporation of a premature stop codon in the reading frame of a strain HS ortholog, independent of the presence of any frameshift resulting from an indel.

a Nonsense mutations are classified as point mutations that catalyze the incorporation of a premature stop codon in the reading frame of an HS ortholog, independent of the presence of any frameshifting indel.

## Discussion

Phylogenetic analysis of strain HS indicates that it shares a close relationship with the *Sodalis*-allied endosymbionts that are found in a wide range of insect hosts, including tsetse flies, weevils, lice and stinkbugs. In terms of 16S rRNA sequence identity, strain HS is most closely related to endosymbionts found in the chestnut weevil, *Curculio sikkimensis* and the stinkbug, *Cantao occelatus*. Interestingly, only limited numbers of these insects maintain *Sodalis*-allied endosymbionts in their natural environment [21]–[23], suggesting that they do not maintain persistent (maternally-transmitted) infections. Furthermore, it is notable that the sequences from strain HS, *C. sikkimensis* and *C. occelatus* are localized on very short branches in our phylogenetic trees, indicating that these particular lineages are evolving slowly in comparison to other *Sodalis*-allied endosymbionts. This low rate of molecular sequence evolution, along with the observation that the strain HS genome shows no sign of the characteristic degenerative changes that are known to accompany the transition to the obligate host-associated lifestyle, leads us to propose that strain HS represents an environmental progenitor of the *Sodalis*-allied clade of insect endosymbionts.

Closely related members of the *Sodalis*-allied clade of insect endosymbionts have now been identified in a wide range of distantly related insect taxa, including some that are known to feed exclusively on plants and others that are known to feed exclusively on animals [8]. Although strain HS was isolated from the wound of a human host, it is difficult to assess the extent of its pathogenic capabilities, due to the fact that antibiotic treatment commenced three days prior to microscopic examination and culturing. In addition, the available evidence indicates that the original source of the infection was a branch from a dead crab apple tree. This implies that strain HS was present either on the bark or in the woody tissue of this tree, possibly acting as a pathogen or saprophyte. Furthermore, it is interesting to note that *C. sikkimensis* and *C. occelatus*, whose symbionts are most closely related to strain HS, are both known to feed on trees [35], [36]. In addition, some wood and bark-inhabiting longhorn beetles, including *Tetropium castaneum* (Figure 1) have recently been found to maintain *Sodalis*-allied endosymbionts [37]. Moreover, the ability of strain HS to persist in both plant and animal tissues is compatible with the observation that diverse representatives of both herbivorous and carnivorous insects have acquired *Sodalis*-allied symbionts.

In a comparative sense, relationships involving the *Sodalis*-allied endosymbionts are considered to be relatively recent in origin. Indeed, evidence of host-symbiont co-speciation only exists in the case of grain weevils, *Sitophilus* spp., which were estimated to have co-evolved with their *Sodalis*-allied endosymbionts for a period of around 20 MY, following the replacement of a more ancient lineage of endosymbionts in these insects [38], [39]. The notion of a recent origin of the *Sodalis*-allied endosymbionts is further supported by the fact that the whole genome sequence of *S. glossinidius* is substantially larger than that of long-established mutualistic insect endosymbionts, and is close to the size of related free-living bacteria [24]. However, the *S. glossinidius* genome does have an unusually low coding capacity resulting from the presence of a large number of pseudogenes [24], [25]. This suggests that *S. glossinidius* is at an intermediate stage in the process of genome degeneration, in which many protein coding genes have been inactivated by indels and nonsense mutations but have not yet been deleted from the genome. In the current study we show that the genome of the grain weevil symbiont, SOPE, is at a similar stage of degeneration as evidenced by the presence of a comparable number of pseudogenes and a large number of repetitive insertion sequence elements.

In a comparative sense, it is interesting to note that SOPE and strain HS share a substantially higher level of sequence similarity, genome-wide, in comparison to *S. glossinidius* and strain HS (Figure 2). In the context of the progenitor hypothesis, the disparity in the relationship between strain HS, SOPE and *S. glossinidius* can be explained by the idea that there may be a substantial level of diversity among free-living relatives of the *Sodalis*-allied symbionts in the environment, and that we simply happened to characterize a representative that is more closely related to the ancestral progenitor of SOPE. While this is likely to be true to some extent, the close relationship between strain HS and SOPE can also be explained by the notion that the SOPE-grain weevil symbiosis has a more recent origin than the *S. glossinidius*-tsetse symbiosis. Our results provide several compelling lines of evidence in support of this idea. Most significantly, we found that the pseudogenes of *S. glossinidius* contain a higher average density of disrupting mutations relative to their counterparts in SOPE. This suggests that the pseudogenes of *S. glossinidius* have been evolving under relaxed selection for a longer period of time, consistent with the hypothesis of a more ancient origin of host association catalyzing the neutralization of these genes. In addition, the genome of SOPE is predicted to have a larger proportion of “cryptic” pseudogenes; genes evolving neutrally that have not yet had sufficient time to accumulate nonsense or frameshifting mutations that disrupt their translation. Finally, it is notable that the GC4 sites of *S. glossinidius* have a higher AT-content than those of strain HS and SOPE (Figure 8). Assuming that the AT-bias at GC4 sites accumulates in a clock-like manner following the onset of the symbiosis, this again supports a more ancient origin for the symbiosis involving *S. glossinidius*.

In the current study, a comparative analysis of the genome sequences of strain HS, SOPE and *S. glossinidius* has provided an unprecedentedly detailed view of the nascent stages of genome degeneration in symbiosis. Taken together, our results indicate that irreversible degenerative changes, including gene inactivation and loss, in addition to base composition bias, commence rapidly following the onset of an obligate relationship. Indeed, the close relationship observed between strain HS and SOPE illustrates the potency of the degenerative evolutionary process at an early stage in the evolution of a symbiotic interaction. This is exemplified by the fact that SOPE is predicted to have lost 55% of its ancestral gene inventory (34% via gene loss and 21% via gene inactivation) in a period of time sufficient to incur a substitution frequency of only 4.3% at the highly variable GC4 sites of intact protein coding genes (Figure 8). Although estimates of genome wide synonymous clock rates vary by several orders of magnitude in bacteria [40], an estimate of *μ*_s_ = 2.2×10^−7^, derived recently for another insect endosymbiont, *Buchnera aphidicola* [41], places the divergence of strain HS and SOPE at only c. 28,000 years, which is much more recent than previous estimates obtained for the origin of the SOPE symbiosis [38], [39].

While the broad distribution of recently derived endosymbionts in phylogenetically distant insect hosts has previously been attributed to interspecific symbiont transfer events [10], [11], the results outlined in the current study indicate that diverse insect species can also acquire novel symbionts through the domestication of bacteria that reside in their local environment. In the case of *S. glossinidius* and SOPE, our comparative analyses support the notion that these symbionts were acquired independently, as evidenced by the presence of distinct mutations in shared pseudogenes. This also implies that symbionts rapidly become specialized towards a given host, likely restricting their abilities to switch hosts. Although the current study highlights the first description of a close free-living relative of the *Sodalis*-allied symbionts, it should be noted that environmental microbial diversity is vastly undersampled [42]. Thus, it is conceivable that close relatives of extant insect endosymbionts, such as strain HS, are widespread in nature and provide ongoing opportunities for a wide range of insect hosts to domesticate new symbiotic associates. Furthermore, since many insects serve as vectors for plant and animal pathogens [43], it is conceivable that mutualistic associations arise as a consequence of the domestication of vectored pathogens. This hypothesis is compelling because such pathogens are not expected to negatively impact the fitness of their insect vectors [44] and under those circumstances the transition to a mutualistic lifestyle could be achieved without any need to attenuate virulence towards the insect host.

## Materials and Methods

### Isolation and Phylogenetic Analysis of Strain HS

Strain HS was isolated on MacConkey agar at 35°C and 5% CO_2_. 16S rRNA and *groEL* sequences were amplified from strain HS using universal primers. Following cloning of PCR products, eight clones were sequenced from each gene and consensus sequences were used in phylogenetic analyses. Sequence alignments were generated for 16S rRNA and *groEL* using MUSCLE [45]. PhyML [46] was then used to construct phylogenetic trees using the HKY85 [47] model of sequence evolution with 25 random starting trees and 100 bootstrap replicates.

### Weevil Cultures and DNA Isolation

Synchronous cultures of *Sitophilus oryzae* and *Sitophilus zeamais* were reared on organic soft white wheat grains and corn kernels respectively, and maintained at 25°C with 70% relative humidity. Bacteriomes (containing the bacterial endosymbionts SOPE and SZPE) were isolated from 5th instar *S. oryzae* and *S. zeamais* larvae by dissection and homogenized at a sub-cellular level to release bacteria from host bacteriocyte cells; bacterial cells were then separated from host cells via centrifugation (2,000×*g*, 5 min). Total genomic DNA was then isolated from bacteria using the Qiagen DNeasy Blood & Tissue Kit (Qiagen, Valencia, CA).

### SOPE and SZPE Shotgun Library Construction

Six mg of genomic DNA was hydrodynamically sheared in 5 mM Tris, 1 mM EDTA, 100 mM NaCl (pH 8) buffer to a mean fragment size of 10 kb. The sample was washed and concentrated by ultrafiltration in a Centricon-100 (Millipore, Billerica, MA) and eluted in 250 µl of 2 mM Tris (pH 8). The fragments were end-repaired by treatment with T4 DNA polymerase (New England Biolab, Beverly, MA) to generate blunt ends. The DNA was then extracted with phenol/chloroform, ethanol precipitated, and 5′ phosphorylated with T4 polynucleotide kinase (NEB). Ten mM of double-stranded, biotinylated oligonucleotide adaptors were blunt-end ligated onto the sheared genomic fragments at room temperature for 25 h using 10,000 cohesive end units of high concentration T4 DNA ligase (NEB). Unligated adaptors were removed by ultrafiltration in a Centricon-100. The adaptored fragments were bound to streptavidin-coated magnetic beads (Invitrogen), and after binding and washing, the adaptored genomic fragments were eluted in 10 mM TE (pH 8). Fragments in the 9.5–11.5 kb size range were gel purified after separation on a 0.7% 1× TAE agarose gel, and the purified DNA was electroeluted from the agarose and desalted by ultrafiltration in a Centricon-100.

### SOPE and SZPE Shotgun Sequencing

pWD42 vector (GenBank: AF129072.1) was linearized by digestion with *Bam*HI (NEB) at 37°C for 4 h, extracted with phenol/chloroform, ethanol precipitated and resuspended in 100 ml of 2 mM Tris (pH 8.0). Ten picomoles of double-stranded, biotinylated oligo adaptors were ligated onto the *Bam*HI-digested vector at 25°C for 16 hrs using 4,000 units of T4 DNA ligase (NEB). Unligated adaptors were removed by ultrafiltration in a Centricon-100. The adaptored vector was bound to streptavidin-coated magnetic beads and the non-biotinylated adaptored vector was eluted in 10 mM TE (pH 8). One hundred ng each of adaptored vector and genomic DNA were annealed without ligase in 10 ml of T4 DNA ligase buffer (NEB) at 25°C for one hour. Two ml aliquots of the annealed vector/insert were transformed into 100 ml of XL-10 chemically competent *E. coli* cells (Agilent Technologies, Santa Lara, CA) and plated on LB agar plates containing 20 µg/ml ampicillin. A total of 23,808 bacterial colonies were picked into 96-well microtiter dishes containing 600 ml of terrific broth (TB)+20 µg/ml ampicillin and grown at 30°C for 16 h. Fifty ml aliquots were removed from the library cultures, mixed with 50 ml of 14% DMSO, and archived at −80°C. The 200 ml cultures were diluted 1∶4 in TB amp and runaway plasmid replication was induced at 42°C for 2.25 h. Plasmid DNA was purified by alkaline lysis, and cycle sequencing reactions were performed with forward and reverse sequencing primers using ABI BigDye v3.1 Terminator chemistry (Applied Biosystems, Foster City, CA). The reactions were ethanol precipitated, resuspended in 15 ul of dH_2_O, and sequence ladders were resolved on an ABI 3730 capillary instrument prepared with POP-5 capillary gel matrix.

### SOPE Genome Sequence Assembly and Finishing

Following elimination of any sequences encoding contaminating plasmid vector or host insect sequences, 38,755 shotgun reads were assembled using the Phusion assembler [48] using the paired-end sequences as mate-pair assembly constraints. Contig assemblies were viewed and edited in Consed [49], and reads with high quality (Phred>20) discrepancies were disassembled. After inspection and manual assembly to extend contigs, gaps were closed by iterative primer walking (895 primer walk sequence reads) and gamma-delta transposon-mediated full-insert sequencing of plasmid clones (6,165 sequence reads across 103 transposed plasmid clones) using an established protocol [50]. The average insert size of the plasmid library in the finished SOPE assembly was found to be 8.2 kb.

### SOPE Fosmid Library Construction and Genome Sequence Validation

The SOPE fosmid library was constructed using the Epicenter EpiFOS Fosmid Library Production Kit (Epicentre Biotechnologies, Madison, WI), using SOPE total genomic DNA. 1,404 paired-end reads were generated from 702 fosmid inserts and mapped onto the assembly derived from the plasmid shotgun sequencing for validation (Figure S2).

### Strain HS Sequencing

Strain HS genomic DNA was isolated from liquid culture using the Qiagen DNeasy Blood & Tissue Kit (Qiagen, Valencia, CA). Five micrograms of total genomic DNA was used to construct a paired-end sequencing library using the Illumina paired-end sample preparation kit (Illumina, Inc. San Diego, CA) with a mean fragment size of 378 base pairs. This library was then sequenced on the Illumina GAIIx platform generating 26,891,485 paired-end reads of 55 bases in length.

### Strain HS Sequence Assembly

Paired-end reads were quality filtered using Galaxy [51], [52] and low quality paired-end reads (Phred<20) were discarded. The remaining 17,054,405 reads were then assembled using Velvet [53] with a k-mer value of 37, with expected coverage of 119 and a coverage cutoff value of 0.296. The resulting assembly consisted of 271 contigs with an N50 size of 231,573 and a total of 5,135,297 bases. No sequences were found to share significant sequence identity with genes encoding plasmid replication functions, suggesting that strain HS does not maintain any extrachromosomal elements.

### Strain HS Annotation

The assembled draft genome sequence of strain HS was annotated by automated ORF prediction using GeneMark.hmm [54]. The annotation was then adjusted manually in Artemis [55] using the published *Sodalis glossinidius* genome sequence [25] as a guide. ORFs were annotated as putatively functional only if (i) their size was ≥90% of the most closely related ORF derived from a free-living bacterium in the GenBank database, and (ii) they did not contain any frameshifting indel(s).

### Genome Alignment, COG Classification, and Computational Analyses

Curation of the strain HS genome sequence was performed in Artemis [55]. ORFs were classified into COG categories using the Cognitor software [56]. Syntenic links shown in Figure 2 were determined by pairwise nucleotide alignments between strain HS contigs and *S. glossinidius* (GenBank: NC_007712.1) or the finished SOPE genome using the Smith-Waterman algorithm as implemented in the cross_match algorithm [49]. Figure 2 was prepared from data obtained from these alignments using CIRCOS [57]. The metrics depicted in Table 1, Table 2, and Figure 8 were computed from pairwise nucleotide sequence alignments of strain HS, *S. glossinidius* and SOPE ORFs using custom scripts. Candidate genes were classified as intact orthologs when their alignment spanned >99% of the HS ORF length (or 90% for ORFs <300 nucleotides in size) and did not contain frameshifting indels or premature stop codons.

### Monte Carlo Pseudogene Simulation

A simple Monte Carlo approach was implemented to simulate the evolution of pseudogenes in *S. glossinidius* and SOPE. The simulation facilitated the progressive accumulation of random mutations in all strain HS orthologs of both intact genes and pseudogenes identified in the current *S. glossinidius* or SOPE gene inventories. Mutations accumulated in proportion to ORF size in a randomly selected class of neutral genes of user-defined size over a defined number of mutational cycles. At preset cycle intervals, the simulation recorded (i) the difference in size between intact and disrupted sequences, (ii) the number of neutral genes that have accumulated one or more disrupting mutations, and (iii) the density of disrupting mutations, which was calculated based on the cumulative size of all neutral genes.

### Accession Numbers

The GenBank accession numbers for sequences used in Figure 1 are as follows: Endosymbiont of *Circulio sikkimensis* 16S rRNA, (AB559929.1), *groEL*, (AB507719); *Vibrio cholerae* 16S rRNA, (NC_002506.1), *groEL*, (NC_002506.1); *Dickeya dadantii* 16S rRNA (CP002038.1), *groEL*, (CP002038.1); *Escherichia coli* 16S rRNA, (NC_000913.2), *groEL*, (NC_000913.2); *Candidatus* Moranella endobia 16S rRNA, (NC_015735), *groEL*, (NC_015735); *Sodalis glossinidius* 16S rRNA, (NC_007712.1), *groEL*, (NC_007712.1); *Yersinia pestis* 16S rRNA, (NC_008150.1), *groEL*, (NC_008150.1); *Wigglesworthia glossinidia* 16S rRNA, (NC_004344.2), *groEL*, (NC_004344.2); *Candidatus* Blochmannia pennsylvanicus 16S rRNA, (NC_007292), *groEL*, (NC_007292); Endosymbiont of *Cantao ocellatus* 16S rRNA, (AB541010), *groEL*, (BAJ08314); Endosymbiont of *Columbicola columbae* 16S rRNA, (AB303387), *groEL*, (JQ063388); *Sitophilus zeamais* primary endosymbiont 16S rRNA, (AF548142), *groEL* (JX444567); *Sitophilus oryzae* primary endosymbiont 16S rRNA, (AF548137), *groEL* (AF005236); Strain HS 16S rRNA, (JX444565), *groEL* (JX444566). The GenBank accession numbers for sequences used in Figure 4 are as follows: Strain HS Figure 4A (JX444569), Figure 4B (JX444571), Figure 4C (JX444572); *Sitophilus oryzae* primary endosymbiont Figure 4A (JX444568), Figure 4B (JX444570), Figure 4C (JX444573).

## Supporting Information

Figure S1 Alignment between strain HS contigs (top) and the chromosome of *Dickeya dadantii*. The draft strain HS contigs are depicted in an arbitrary color scheme (outer top ring). On the upper track, grey bars depict genes unique to strain HS whereas green bars depict strain HS genes that share orthologs with the aligned *D. dadantii* chromosome. On the lower track, green and red bars represent intact and disrupted genes (respectively) in the *D. dadantii* chromosome, and blue bars indicate prophage and IS-element ORFs.
(TIF)

Figure S2 Genome assembly validation of the 4.5 Mb SOPE genome using plasmid and fosmid mate-pair coverage. The finished SOPE chromosome sequence assembly was obtained by paired-end plasmid shotgun sequencing, primer walking and directed transposon-based full-insert plasmid sequencing. The physical map of the plasmid paired-ends are represented in blue. Clones from underrepresented regions and IS-element clusters were completely sequenced by transposon-mediated sequencing (red; see Materials and Methods). The resulting finished assembly (circular chromosome: 4,513,139 bp) was validated by fosmid paired-end sequencing (orange). Depth of plasmid clone physical coverage is depicted in the histogram (yellow). The locations of the four most abundant families of IS-elements are depicted by the inner bars (red, IS903; blue, IS256; green, IS21; purple, ISL3).
(TIF)

Table S1 List of complete strain HS gene products and status of orthologs in *S. glossinidius* and SOPE. Candidate virulence genes are highlighted in the column labeled “V”, according to the presence of orthologs in animal (A) or plant (P) pathogens.
(XLSX)
