# Supplementary figures and images for: A Novel Human-Infection-Derived Bacterium Provides Insights into the Evolutionary Origins of Mutualistic Insect–Bacterial Symbioses
Source: PLoS Genet. 2012 Nov 15;8(11):e1002990. doi: 10.1371/journal.pgen.1002990 (PMC3499248; doi:10.1371/journal.pgen.1002990)

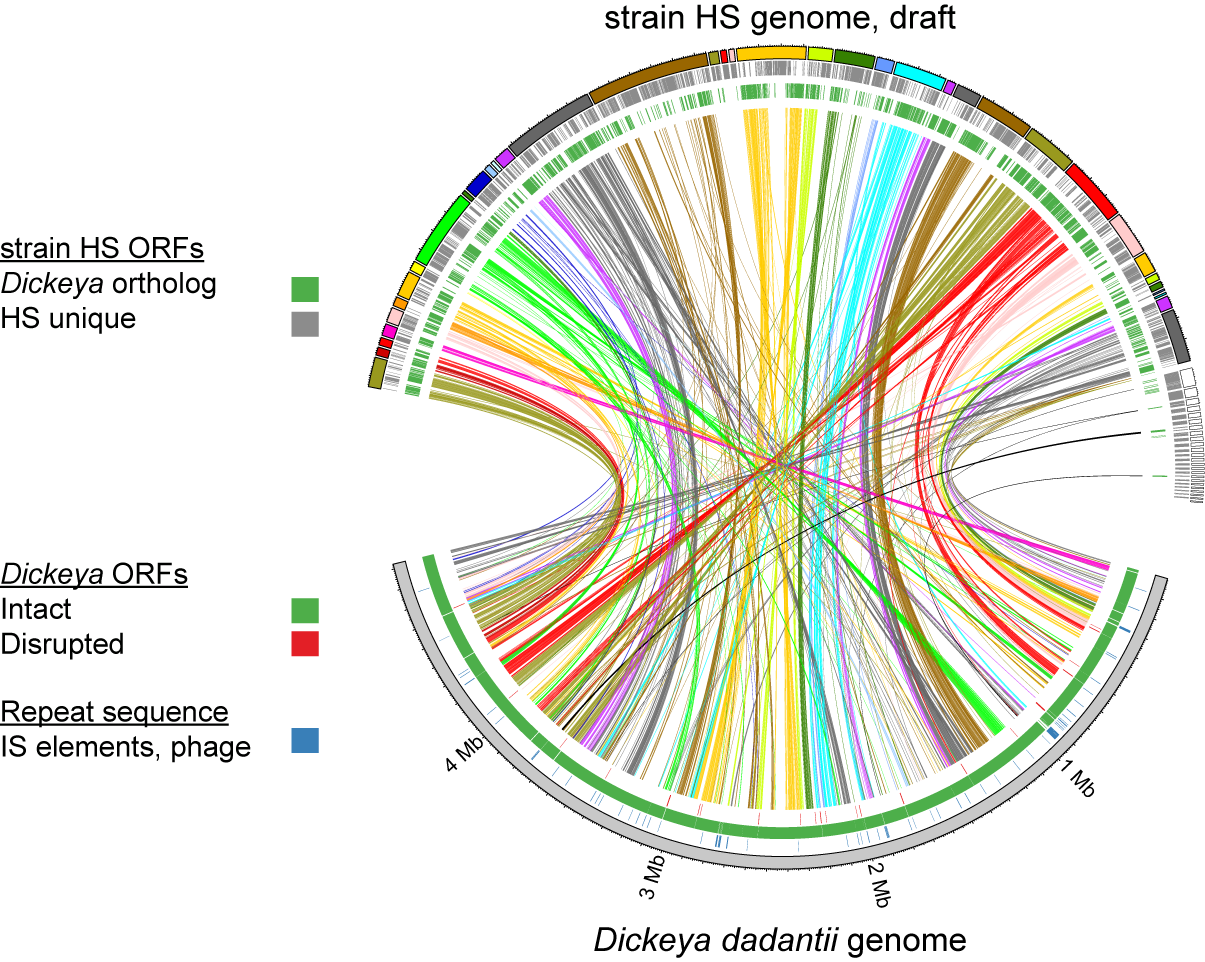

Supplement: Figure S1 — Alignment between strain HS contigs (top) and the chromosome of Dickeya dadantii. The draft strain HS contigs are depicted in an arbitrary color scheme (outer top ring). On the upper track, grey bars depict genes unique to strain HS whereas green bars depict strain HS genes that share orthologs with the aligned D. dadantii chromosome. On the lower track, green and red bars represent intact and disrupted genes (respectively) in the D. dadantii chromosome, and blue bars indicate prophage and IS-element ORFs. (TIF) [file pgen.1002990.s001.tif]
